# Morphological Control of Crystalline Savolitinib via the Volatile Deep Eutectic Solvent Technique

**DOI:** 10.1021/acs.cgd.4c00035

**Published:** 2024-03-01

**Authors:** Jasmine. E. Symons, Charlie Hall, James F. McCabe, Simon R. Hall

**Affiliations:** † Complex Functional Materials Group, School of Chemistry, University of Bristol, Bristol BS8 1TS, U.K.; ‡ Complex Functional Materials Group, School of Chemistry, University of Bristol, Bristol BS8 1TS, United Kingdom; Centre for Doctoral Training in Condensed Matter Physics, HH Wills Physics Laboratory, Bristol BS8 1TL, U.K.; § Early Product Development and Manufacturing, Pharmaceutical Sciences, R&D, AstraZeneca, Macclesfield SK10 2NA, U.K.; ∥ Complex Functional Materials Group, School of Chemistry, University of Bristol, Bristol BS8 1TS, U.K.

## Abstract

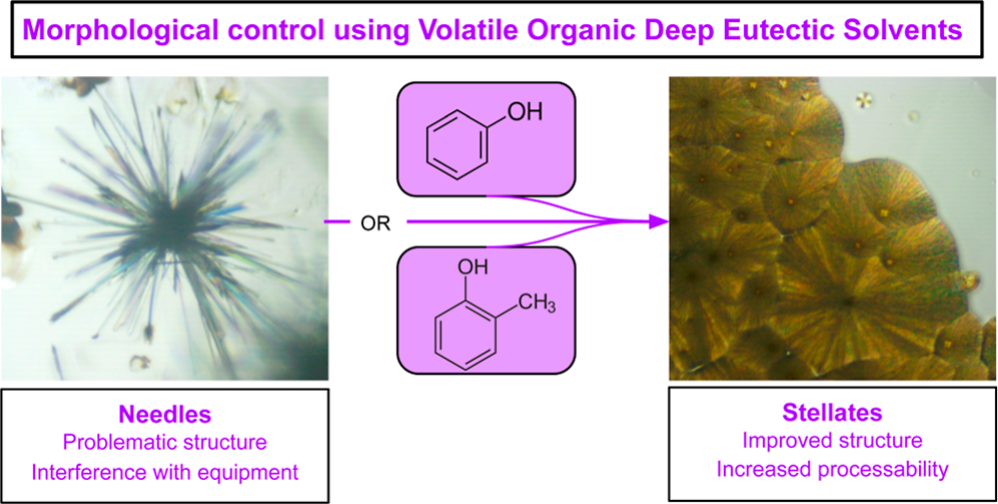

Savolitinib is a compound that can crystallize in an undesirable, high aspect ratio needle morphology. This morphology type may cause issues in downstream processing. This paper demonstrates a unique method to alter the crystal morphology of savolitinib to make it more processable, resulting in the active pharmaceutical ingredient (API) crystallizing out in considerably more processable stellates. The volatile deep eutectic solvent technique presents a simple and scalable method for changing the crystal morphology while maintaining the polymorph of the API in this case, confirmed via powder X-ray diffraction and differential scanning calorimetry analysis.

## Abstract 2

Savolitinib is a compound that can crystallize in an undesirable, high aspect ratio needle morphology. This work shows a unique method to alter the crystal morphology of savolitinib to more processable stellate structures.

## Introduction

Savolitinib is a highly potent, selective cMET kinase inhibitor used for the treatment of adenocarcinoma, nonsmall cell lung cancer (NSCLC), and renal cell carcinoma in development by AstraZeneca and HUTCHMED (China) Ltd.^1−4^ It has shown success in phase I clinical trials in combination with osimertinib for patients with NSCLC, with a response rate of 48–64%.^5^ It is currently being assessed in phase II clinical trials in patients with adenocarcinoma and sarcomatoid lung cancer with an overall response rate of 47.5% and an average progression-free survival of 6.8 months.^6^ Savolitinib has been temporarily approved for use in China for patients with NSCLC with METex14-skipping alterations that no longer respond to more traditional chemotherapies or are unable to receive chemotherapy.^7^

There are various morphologies in which compounds have the potential to crystallize, one of the more frequent forms being needles.^8,9^ There are a number of factors that can cause a compound to crystallize in this manner,^10,11^ such as the choice of solvent, supersaturation, and the presence of impurities.^10^ Needle-like crystals are an issue in the downstream processing of many active pharmaceutical ingredients (APIs) as they tend to block equipment, are problematic to filter, and have a low packing density.^12,13^ These problems can be expensive and time-consuming to fix, occasionally leading to complete shutdowns of the manufacturing process in order to correct stoppages.^14^ There have been several previous attempts to prevent needle crystallization such as high-shear ultralow attrition agitation, which consists of spinning the crystallizing solution in alternate directions every 3 s^15^ or the addition of additives.^16^ However, many techniques used to circumvent needle growth are usually nonchemical, energy-intensive methods such as thermal cycling^17^ or mechanical, such as milling.^11,18^ Since savolitinib’s development in 2017, however, there has been an effort to overcome processability challenges with the drug as that it invariably crystallizes in an undesirable, high aspect ratio needle morphology. One method used to attempt to control the morphology of savolitinib was via the application of an external magnetic field to encourage magnetically aligned growth. Although there were slight deviations from the needle-like growth, with the crystals growing perpendicular to the magnetic field, the overall morphology was still needle-like.^19^ In each of the preceding cases, the extra steps employed in order to prevent crystals from growing as needles add to the overall cost of taking an API to market. What is required, therefore, is a method by which an API that grows in a needle morphology naturally and spontaneously crystallizes in a more processable form.

One possible method could be through the use of deep eutectic solvents (DESs). DESs are a subset of ionic liquids (ILs), defined as “liquids close to the eutectic composition of the mixtures, i.e., the molar ratio of the components which gives the lowest melting point”.^20,21^ They are a combination of Lewis or Brønsted acids and bases containing various anionic and cationic species.^20,21^ They differ from ILs because of their combination of anions and cations, whereas ILs tend to contain just one type of discrete anion or cation.^20,21^ For DESs, when two or more molecular species are bought together, they extensively hydrogen bond, leading to a depression in the melting point of the mixture.^22^ It is the interaction between the hydrogen bond donor and acceptor that leads to their stability.^22^ They can be seen as a greener alternative to many solvents, especially ILs, due to simple synthesis, low volatility, low cost, and high biodegradability.^23^ A recent development in DESs that is pertinent to the control of API morphology was the discovery of a new class of DESs, where one of the components of the liquid was volatile at room temperature and pressure (RTP). When the volatile component leaves the DES system, the “reversal of the eutectic liquification”^22^ allows for polymorphic and morphological control in crystal growth, as well as aiding the formation of cocrystals.^22^ The development of these volatile DESs (VODES) holds significant potential for controlled crystal growth at RTP that is scalable for use in the pharmaceutical industry.

Here, we show that the recently discovered VODES technique, when applied to the crystal growth of savolitinib, effectively controls the morphology, restricting needle growth, so the crystals do not present as free forming needles but instead as spherical bundles in stellate structures which are typically more industrially processable at RTP and without the need for any additives. Using two different solvents (either phenol or *o*-cresol) at molar ratios varying from 15:1, 20:1, and 25:1, the morphological structure savolitinib was controlled to form confined stellates rather than needles. This was then analyzed using optical microscopy, differential scanning calorimetry (DSC), and powder X-ray crystallography (PXRD).

## Materials and Methods

Experiments with VODES were performed according to Potticary et al.^22^ The sample of savolitinib was provided by AstraZeneca. Briefly, the crystallization of savolitinib was explored at molar ratios of 15:1, 20:1, and 25:1 VODES/savolitinib in phenol and *o*-cresol. Savolitinib crystallization usually required around 3–4 days in a 50 °C oven and resulted in light yellow stellate structures. All amounts of VODES and the masses of savolitinib used for each experiment are shown (Table 1).

**Table 1:** Amounts of Solvents Used for Each Ratio of Savolitinib Solution and the Amount of Savolitinib Added for Each Ratio

ratio	solvent (mol)	cresol (mg)	cresol (ml)	phenol (mg)	savolitinib (mg)
15:1	0.001	108.1	0.1030	94.11	17.27
20:1	0.001	108.1	0.1030	94.11	23.02
25:1	0.001	108.1	0.1030	94.11	34.54

Control samples consisted of the crystallization of savolitinib out of ethanol at a concentration of 8 mg mL^–1^. 16 mg of savolitinib was added to a 5 mL vial with 2 mL of ethanol. This was allowed to dissolve in a 50 °C oven for 2 h to form a colorless solution. This solution was then transferred to a watch glass, and the ethanol was allowed to evaporate off for 24 h, yielding beige, needle-like crystals. These were then removed from the watch glass, crushed, and analyzed using PXRD.

### Powder X-ray Diffraction

PXRD patterns obtained from this project were collected using a Bruker D8 powder diffractometer using Cu Kα (λ = 1.5418 Å) with a PSD LynxEye Detector. Instrumental parameters are as follows: 2θ angle range 5–50°, counting time 1 s per step, and counting step (2θ) 0.01°. Analyzed samples were removed from the microscope slide and then crushed using a pestle and mortar to attain a fine powder. This was then pressed onto a low-background silicon sample holder with a glass slide to ensure that the sample was flat and parallel to the holder for analysis.

### Differential Scanning Calorimetry

DSC analyses was performed using the Discovery 25 with a cooling system. The DSC heaters were purged with nitrogen. The samples were examined in nonhermetically sealed aluminum pans. A small mass of crushed crystals (∼2–10 mg) was added to the nonhermetic pan and sealed using a press.

For savolitinib, samples were heated from 25 to 300 °C, cycled down to −50 °C, and then back up to 300 °C at 10 °C/min. The instrument was calibrated via the pure indium standard.

### Optical Microscopy

All optical microscopy images were obtained using a J. Swift & Son optical microscope with a magnification of 4×. Images were captured using a Brunel Digital Eyecam and analyzed using ToupView.

### Digital Images

Digital images were taken on a Nikon D7200 Digital SLR with a Sigma 105 mm F2.8 EX DG OS HSM Macro Lens.

## Results and Discussion

Initially, it is important to clarify that it is known that savolitinib and phenol or *o*-cresol in combination form a eutectic mixture because of the previous research carried out with these solvents. Although it was clear, a eutectic mixture was formed with savolitinib and phenol due to the fact that a liquid was formed at RTP when these were combined at ratios ranging from 15:1 to 25:1; this is also supported by the initial study on VODES in 2020.^22^ Such observation could not be made with *o*-cresol as it is already a liquid at RTP, but a study by Yao et al. shows that all isomers of cresol formed DESs with choline chloride, which supports the formation of a eutectic solvent with savolitinib.^24^

In order to confirm that the same polymorph was crystallized, an important consideration to ensure that the savolitinib formed was still relevant to therapeutic use, it was analyzed using PXRD and DSC.

**Figure 1:**
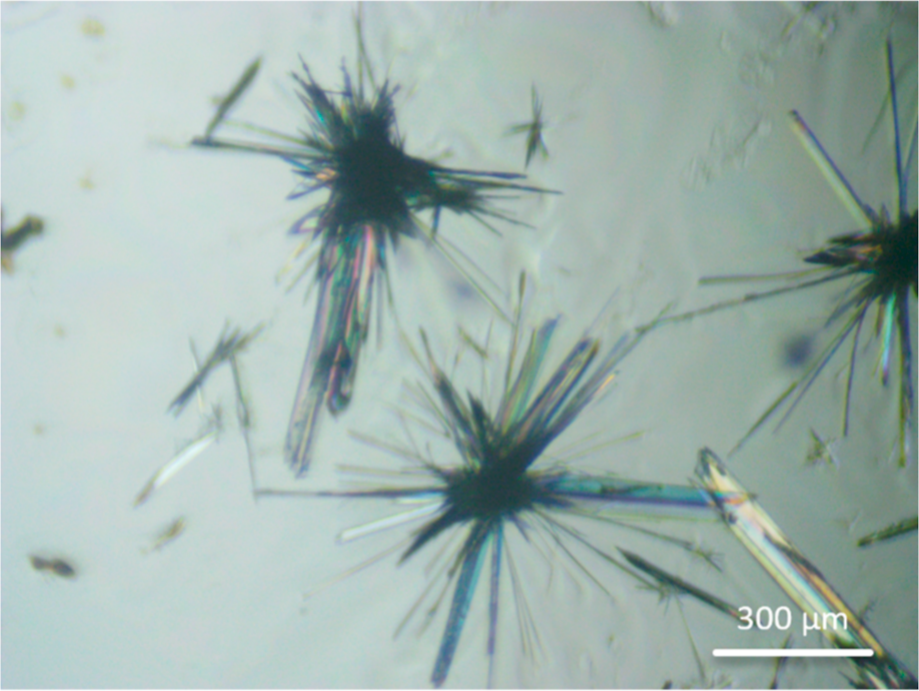
Optical microscopy image of the needle-like structure of savolitinib crystallized from ethanol.

**Figure 2:**
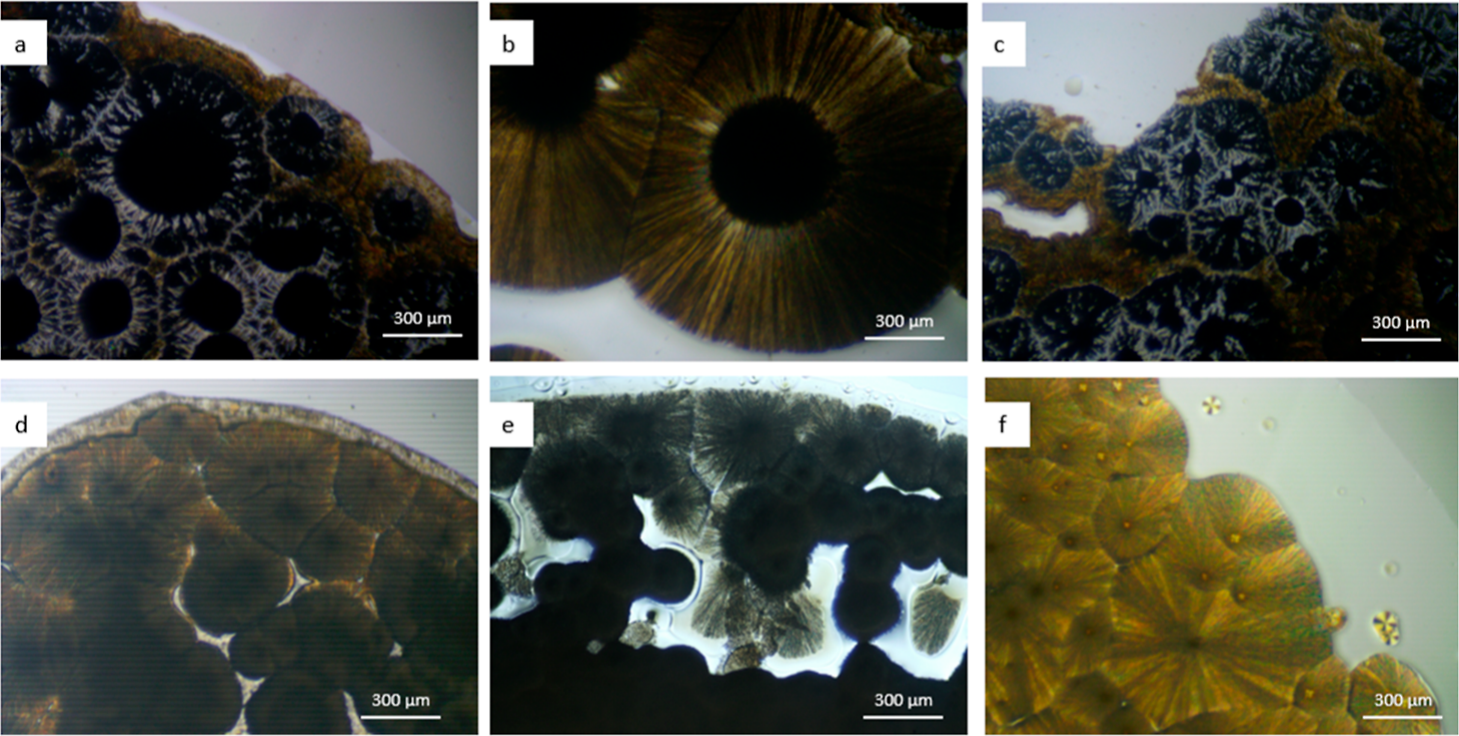
Optical microscopy images of savolitinib crystallized from phenol or *o*-cresol at varying ratios: (a) phenol and savolitinib, 15:1; (b) phenol and savolitinib, 20:1; (c) phenol and savolitinib, 25:1; (d) *o*-cresol and savolitinib, 15:1; (e) *o*-cresol and savolitinib, 20:1; and (f) *o*-cresol and savolitinib, 25:1.

Figure 1 shows the optical microscopy image of a control experiment with savolitinib crystallized from ethanol (8 mg mL^–1^). Large needle structures can be seen forming from *a* point in the center. In contrast, the crystallization of savolitinib from VODES produces acicular crystallites which are instead constrained to flat stellates (Figure 2).

**Figure 3:**
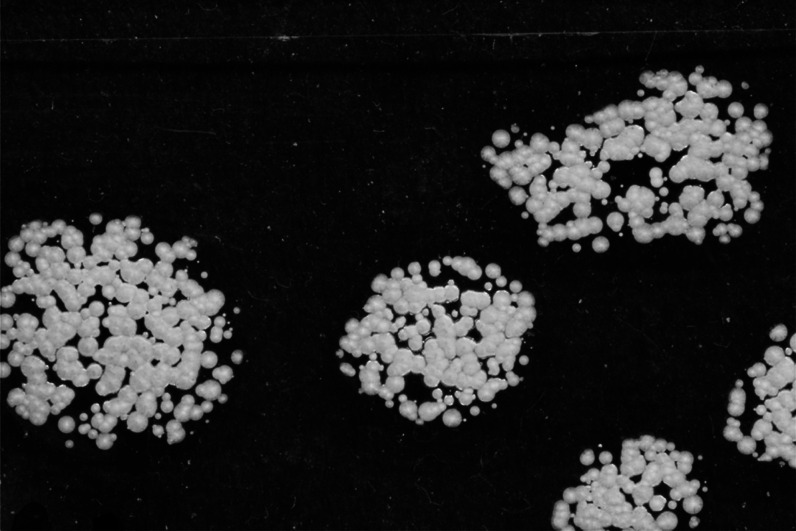
Nikon D7200 digital image of savolitinib crystallized from o-cresol at a ratio of 20:1.

Figure 2 shows optical microscopy images of savolitinib crystallized from phenol and *o*-cresol at various ratios. There is a clear lack of needle growth, with the crystals forming more of a stellate/Maltese cross-structure. The growth of these crystals is reminiscent to that of the needles grown from ethanol, in that they arise from a nucleation point and grow outward isotropically. However, the amorphous nature of the recrystallization from VODES seen by the glassy spots surrounding the stellates in Figure 2 and not seen in recrystallization from ethanol appears to be restricting the savolitinib from growing unconstrained as nonaligned needles. It is interesting to note that different ratios do not yield different morphologies, but different solvents do, presumably due to their different intermolecular interactions. Crystallization from the VODES technique tends to yield more amorphous structures due to the rapidity of evaporation of the volatile component, putting the system under kinetic control. The molecules of the API are therefore locked in a more random, disordered arrangement when evaporation is fast, leading to higher levels of amorphicity.^25^ This amorphicity can be seen in the optical image in Figure 3. Specifically in the central crystallized mass, the glassy sheen of the amorphous character of the crystallization can be seen around the stellates. This suggests that the amorphous solid could be preventing the stellates from forming into well-defined single-crystal needles, by surrounding them in an amorphous shell. These stellates are usually <1.4 mm in diameter compared to the needles grown from ethanol which were usually <1 mm in length. These shell structures are significantly more amenable to downstream processing and can be milled without producing needles.

It is important to note that this increased amorphicity within savolitinib, which led to the changes in morphology, is likely to be accompanied by an increase in solubility. The amorphous content of savolitinib could also crystallize further over time, leading to increased morphological changes. This could affect the pharmacokinetics of the API, so dissolution measurements would have to be carried out to investigate the impact on the rate and extent of drug absorption/uptake. Research is currently ongoing to assess the exact level of solubility increase and therefore overall uptake and bioavailability change when changing the levels of amorphicity of an API.^26^

**Figure 4:**
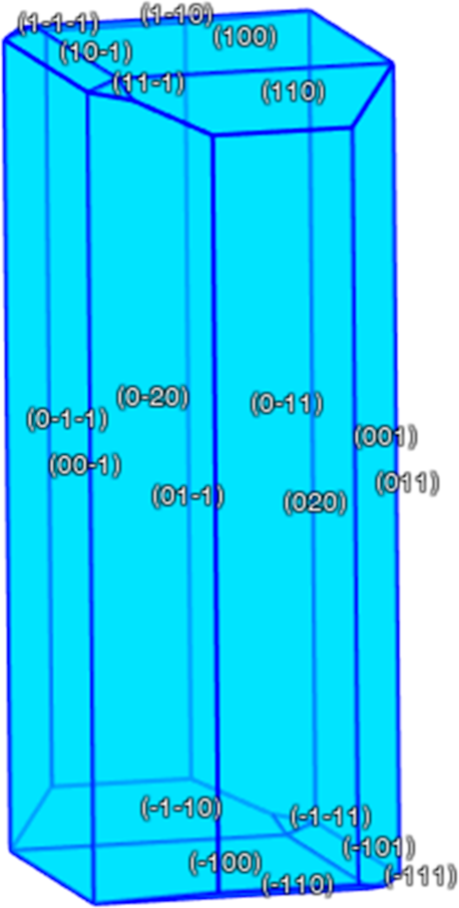
BFDH morphology of the common crystallographic form of savolitinib, highlighting the expected needle-like growth.

An interesting difference can be seen in the morphology of savolitinib based on the volatile component used in the creation of the VODES. When the volatile component was phenol, crystal growth usually resulted in larger stellates, with vein-like projections from the central core of each stellate. The core was invariably larger and denser than the crystals formed from evaporation from *o*-cresol. The stellates formed from *o*-cresol tended to have more closely packed projections from the center, forming more characteristic stellate structures. It is likely that this slight difference in morphology is due to different interactions between the solvent and the savolitinib molecule. One way this can be modeled is using BFDH theory.^27^ BFDH theory states that the crystal growth rate is proportional to 1/*d*_*hkl*_, where *d*_*hkl*_ is the interplanar spacing between each crystal plane in the lattice.^27^ Using the spacing between each plane, we can predict the morphology of a crystal. Obviously, there are limitations to this theory: only one equilibrium crystal morphology is possible, and this does not take into account external factors such as the solvent used and the rate of evaporation. However, BFDH did successfully predict the needle-like structure seen in the savolitinib crystallized from ethanol, shown in Figure 4.

In order to use BFDH to predict how savolitinib is interacting with either phenol or *o*-cresol, a single crystal of a solvate would have to be made, which has a few complications such as difficulties yielding a single crystal and synthesizing a solvate. SCXRD would have to be undertaken in order to obtain a .cif file. This .cif file can be used to run BFDH. Without this, more complicated molecular dynamics simulations would have to be carried out. The energies associated with the growth on the surface of various faces of savolitinib could then be analyzed to explain why the differences in morphologies between ethanol and VODES are occurring as well as between phenol and *o*-cresol.^28^ This would be an interesting avenue of future work and would provide deeper insights into the mechanism of the VODES technique.

X-ray crystallography was used during the analysis of the changing morphology of savolitinib to identify if there were any polymorphic changes within the crystal. There are four known forms of savolitinib, I, II, III, and IV.^29^ Commercial savolitinib is form I,^29^ so it was important to ensure that the same polymorph was present since the morphology when crystallized from VODES is dissimilar. Characteristic peaks for form I savolitinib are 9.5, 11.3, 13.6, 15.3, 16.3, 18.6, 19.1, 22.4, 23.0, and 26.3° 2-theta.

**Figure 5:**
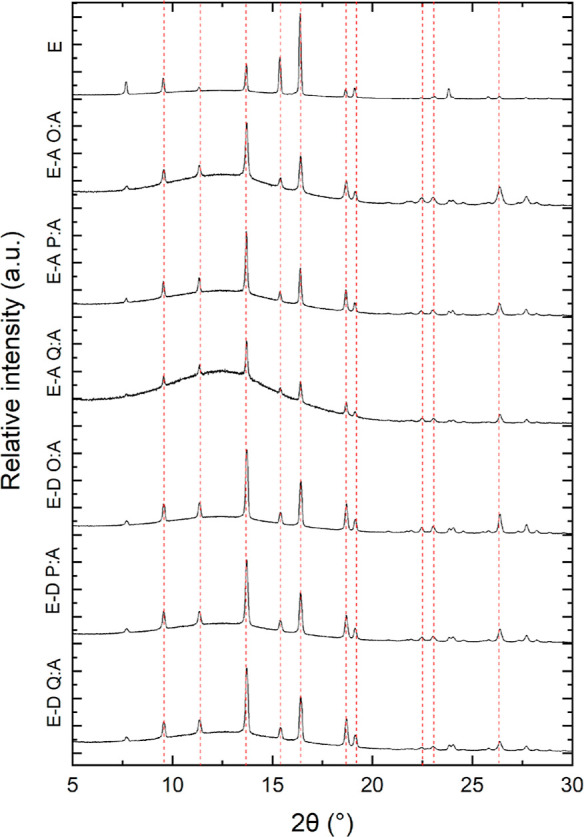
Powder X-ray diffraction patterns for all solvents and ratios of savolitinib showing the same polymorph, form I.

It can be seen from Figure 5 that all samples of savolitinib yielded the form I polymorph, despite the ratio 10:1–25:1 and solvent used (either phenol or *o*-cresol). All of the characteristic peaks of savolitinib are indicated by red dotted lines.

An obvious difference in these patterns is that the baseline can be seen to be shifted upward in the diffraction patterns of savolitinib crystallized from VODES. This is because of the more amorphous nature of the savolitinib crystals, which increases the disorder. This leads to a broad hump in the baseline because of the decrease of order within the crystal lattice compared to a completely crystalline structure.

**Figure 6:**
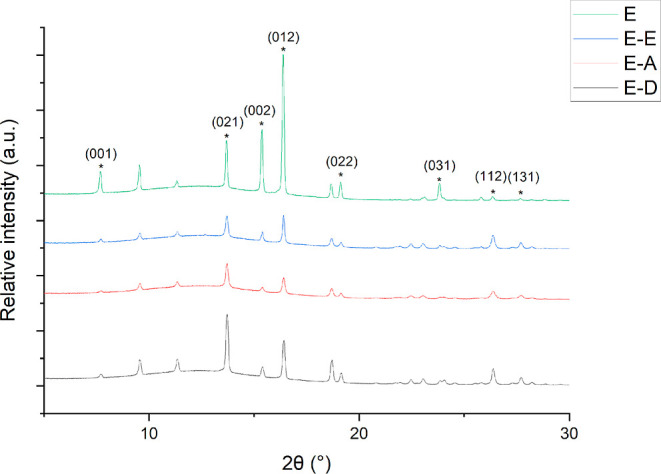
Indexed peaks in PXRD for changes in relative intensity of native savolitinib (E) compared to savolitinib crystallized from ethanol (E–E, 8 mg mL^–1^), savolitinib crystallized from phenol (E–A), and *o*-cresol (E–D) at a 15:1 ratio.

Another interesting observation is the changes in the relative intensities within PXRD of native savolitinib compared to savolitinib crystallized from various solvents. These solvents are ethanol, which is used in simple solvent evaporation acting as a control, and then phenol and *o*-cresol, which are both VODES. This can be seen in Figure 6. A clear increase in peak intensity suggests passivation of certain faces of the crystal, passivation essentially meaning the “coating” of a face with a solvent and constraining its outward growth.^30,31^ An increase in relative peak intensity when savolitinib was evaporated from solvents can be seen on faces (021), (112), and (131), suggesting that these faces become passivated via preferential interactions with the solvent. All other crystal faces show a decrease in peak intensity compared with native savolitinib, suggesting that these faces are interacting less strongly with the solvent.

Since a similar pattern in the relative peak intensities can be seen in all the powder patterns where savolitinib had been dissolved and evaporated in solvents (E–E, E–A, and E–D), this suggests that these solvents are all interacting with the faces indexed in Figure 6 in a similar way. This is somewhat unsurprising since ethanol, phenol, and *o*-cresol all contain hydroxyl groups with the ability to hydrogen bond with certain functional groups expressed on these faces of savolitinib. DSC data also support the fact that all samples of savolitinib crystallized via VODES are of the same polymorph.

**Figure 7:**
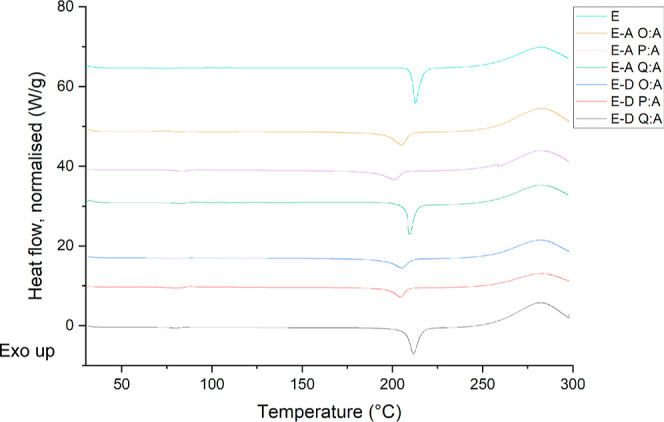
DSC curves for native savolitinib (E) followed by savolitinib crystallized from phenol (A) and *o*-cresol (D) at various ratios ranging between 15:1 and 25:1.

The DSC curves in Figure 7 show a melt transition (*T*_m_) at around 213 °C. However, there is a small variation in melting points in all the samples evaporated from VODES. It seems that the broad trend followed has the lowest melting point at ratios 15:1 and the highest at 25:1; however, this difference is relatively minor. There is a slight variation in *T*_m_ throughout the samples, but it is likely that this is due to the amount of sample used in the DSC experiment. Polymorphs very commonly tend to have different melting points due to the difference in intermolecular interactions between molecules in the crystal lattice, but since all the polymorphs are the same, it is unlikely to be causing the slight differences in melting points in Figure 7.^32^ It would be sensible to suggest that there may be a residual solvent present in the sample, altering the melting point marginally. There is also a very small glass transition (*T*_g_) seen in the curves for savolitinib crystallized from VODES at ∼135 °C. This may be the amorphous portion of the crystal transitioning from solid glass to a more rubbery state and supports the fact that savolitinib is semicrystalline when in the solid state.^33^ Overall, the similar transitions within the DSC curves varying the solvent and the ratio prove that the polymorph of savolitinib yielded is the same. This is also supported by the PXRD patterns matching significantly with that of form I literature data.^29^

## Conclusions

For savolitinib, the VODES technique presented an excellent method to significantly change the morphology of the API from needles to stellates without altering its polymorph. The kinetic crystallization of VODES led to increased amorphous character within the compound, restricting savolitinib from crystallizing out as free forming needles. This has significant industrial relevance for avoiding challenges with downstream processing since the methodology is a simple, cost-effective, and accessible method to prevent crystallization as needle-like structures. PXRD and DSC were used to confirm that the polymorph of savolitinib remained form I.

## Abbreviations

BFDH: Bravais–Freidel–Donnay–Harker
API: active pharmaceutical ingredient
DES: deep eutectic solvent
VODES: volatile deep eutectic solvents
RTP: room temperature and pressure
ILs: ionic liquids
HBD: hydrogen bond donor
HBA: hydrogen bond acceptor
PXRD: powder X-ray diffraction
cMET: mesenchymal-epithelial transition factor
NSCLC: nonsmall cell lung cancer
DSC: differential scanning calorimetry
*T*_m_: melt transition
*T*_g_: glass transition

## References

[ref1] Bauer J.; Spanton S.; Henry R.; Quick J.; Dziki W.; Porter W.; Morris J. Ritonavir: an extraordinary example of conformational polymorphism. Pharm. Res. 2001, 18, 859–866. 10.1023/A:1011052932607. 11474792

[ref2] Shuter J. Lopinavir/ritonavir in the treatment of HIV-1 infection: a review. Ther. Clin. Risk Manage. 2008, 4, 1023–1033. 10.2147/TCRM.S3285. PMC2621403 19209283

[ref3] Reungwetwattana T.; Liang Y.; Zhu V.; Ou S.-H. I. The race to target MET exon 14 skipping alterations in non-small cell lung cancer: The Why, the How, the Who, the Unknown, and the Inevitable. Lung Cancer 2017, 103, 27–37. 10.1016/j.lungcan.2016.11.011. 28024693

[ref4] Markham A. Savolitinib: First Approval. Drugs 2021, 81, 1665–1670. 10.1007/s40265-021-01584-0. 34455538

[ref5] Choueiri T. K.; Heng D. Y. C.; Lee J. L.; Cancel M.; Verheijen R. B.; Mellemgaard A.; Ottesen L. H.; Frigault M. M.; L’Hernault A.; Szijgyarto Z.; Signoretti S.; Albiges L. Efficacy of Savolitinib vs Sunitinib in Patients With MET-Driven Papillary Renal Cell Carcinoma: The SAVOIR Phase 3 Randomized Clinical Trial. JAMA Oncol. 2020, 6, 1247 10.1001/jamaoncol.2020.2218. 32469384 PMC7260692

[ref6] Lu S.; Fang J.; Li X.; Cao L.; Zhou J.; Guo Q.; Liang Z.; Cheng Y.; Jiang L.; Yang N.; Han Z.; Shi J.; Chen Y.; Xu H.; Zhang H.; Chen G.; Ma R.; Sun S.; Fan Y.; Li J.; Luo X.; Wang L.; Ren Y.; Su W. Once-daily savolitinib in Chinese patients with pulmonary sarcomatoid carcinomas and other non-small-cell lung cancers harbouring MET exon 14 skipping alterations: a multicentre, single-arm, open-label, phase 2 study. Lancet Respir. Med. 2021, 9, 1154–1164. 10.1016/S2213-2600(21)00084-9. 34166627

[ref7] Sequist L. V.; Han J.-Y.; Ahn M.-J.; Cho B. C.; Yu H.; Kim S.-W.; Yang J. C.-H.; Lee J. S.; Su W. C.; Kowalski D.; Orlov S.; Cantarini M.; Verheijen R. B.; Mellemgaard A.; Ottesen L.; Frewer P.; Ou X.; Oxnard G. Osimertinib plus savolitinib in patients with EGFR mutation-positive, MET-amplified, non-small-cell lung cancer after progression on EGFR tyrosine kinase inhibitors: interim results from a multicentre, open-label, phase 1b study. Lancet Oncol. 2020, 21, 373–386. 10.1016/S1470-2045(19)30785-5. 32027846

[ref8] Grof Z.; Schoellhammer C. M.; Rajniak P.; Štĕpánek F. Computational and experimental investigation of needle-shaped crystal breakage. Int. J. Pharm. 2011, 407, 12–20. 10.1016/j.ijpharm.2010.12.031. 21232586

[ref9] Mirza S.; Miroshnyk I.; Heinämäki J.; Antikainen O.; Rantanen J.; Vuorela P.; Vuorela H.; Yliruusi J. Crystal morphology engineering of pharmaceutical solids: tabletting performance enhancement. AAPS PharmSciTech 2009, 10, 113–119. 10.1208/s12249-009-9187-4. 19184449 PMC2663677

[ref10] Lovette M. A.; Doherty M. F. Needle-Shaped Crystals: Causality and Solvent Selection Guidance Based on Periodic Bond Chains. Cryst. Growth Des. 2013, 13, 3341–3352. 10.1021/cg301830u.

[ref11] Eren A.; Szilagyi B.; Quon J. L.; Papageorgiou C. D.; Nagy Z. K. Experimental Investigation of an Integrated Crystallization and Wet-Milling System with Temperature Cycling to Control the Size and Aspect Ratio of Needle-Shaped Pharmaceutical Crystals. Cryst. Growth Des. 2021, 21, 3981–3993. 10.1021/acs.cgd.1c00308.

[ref12] Lovette M. A.; Browning A. R.; Griffin D. W.; Sizemore J. P.; Snyder R. C.; Doherty M. F. Crystal Shape Engineering. Ind. Eng. Chem. Res. 2008, 47, 9812–9833. 10.1021/ie800900f.

[ref13] Puel F.; Verdurand E.; Taulelle P.; Bebon C.; Colson D.; Klein J.; Veesler S. Crystallization mechanisms of acicular crystals. J. Cryst. Growth 2008, 310, 110–115. 10.1016/j.jcrysgro.2007.10.006.

[ref14] Civati F.; O’Malley C.; Erxleben A.; McArdle P. Factors Controlling Persistent Needle Crystal Growth: The Importance of Dominant One-Dimensional Secondary Bonding, Stacked Structures, and van der Waals Contact. Cryst. Growth Des. 2021, 21, 3449–3460. 10.1021/acs.cgd.1c00217. PMC8273860 34267600

[ref15] Liu Y.; Lai W.; Yu T.; Ma Y.; Kang Y.; Ge Z. Understanding the growth morphology of explosive crystals in solution: insights from solvent behavior at the crystal surface. RSC Adv. 2017, 7, 1305–1312. 10.1039/C6RA26920F.

[ref16] Yu W.; Liao L.; Bharadwaj R.; Hancock B. C. Prediction of Powder Flow of Pharmaceutical Materials from Physical Particle Properties Using Machine Learning. Powder Technol. 2017, 313, 1–8. 10.1016/j.powtec.2017.02.043.

[ref17] Wu Z.; Yang S.; Wu W. ‘Application of temperature cycling for crystal quality control during crystallization’. CrystEngComm 2016, 18, 2222–2238. 10.1039/c5ce02522b.

[ref18] Lechuga-Ballesteros D.; Rodríguez-Hornedo N. The influence of additives on the growth kinetics and mechanism of l-alanine crystals-alanine crystals. Int. J. Pharm. 1995, 115, 139–149. 10.1016/0378-5173(94)00216-R.

[ref19] Hall C. unpublished work.

[ref20] Smith E. L.; Abbott A. P.; Ryder K. S. Deep Eutectic Solvents (DESs) and Their Applications. Chem. Rev. 2014, 114, 11060–11082. 10.1021/cr300162p. 25300631

[ref21] Abranches D. O.; Coutinho J. A. Everything You Wanted to Know about Deep Eutectic Solvents but Were Afraid to Be Told. Annu. Rev. Chem. Biomol. Eng. 2023, 14, 141–163. 10.1146/annurev-chembioeng-101121-085323. 36888992

[ref22] Potticary J.; Hall C.; Hamilton V.; McCabe J. F.; Hall S. R. Crystallization from Volatile Deep Eutectic Solvents. Cryst. Growth Des. 2020, 20, 2877–2884. 10.1021/acs.cgd.0c00399.

[ref23] Hou Y.; Yao C.; Wu W. Deep Eutectic Solvents: Green Solvents for Separation Applications. Acta Phys.-Chim. Sin. 2018, 34, 873–885. 10.3866/pku.whxb201802062.

[ref24] Yao C.; Liu H.; Wu H.; Song X.; Wang X.; Ren S.; Wu W. Comparative study on the deep eutectic solvents formed by choline chloride and cresol isomers from theoretical and experimental perspectives. J. Mol. Liq. 2022, 367, 120420 10.1016/j.molliq.2022.120420.

[ref25] Liu Y.; Gabriele B.; Davey R. J.; Cruz-Cabeza A. J. Concerning Elusive Crystal Forms: The Case of Paracetamol. J. Am. Chem. Soc. 2020, 142, 6682–6689. 10.1021/jacs.0c00321. 32216346

[ref26] Schittny A.; Huwyler J.; Puchkov M. Mechanisms of increased bioavailability through amorphous solid dispersions: a review. Drug Deliv. 2020, 27 (1), 110–127. 10.1080/10717544.2019.1704940. 31885288 PMC6968646

[ref27] Punzo F. Unveiling the role of molecular interactions in crystal morphology prediction. J. Mol. Struct. 2013, 1032, 147–154. 10.1016/j.molstruc.2012.08.010.

[ref28] Rosbottom I.; Ma C. Y.; Turner T. D.; O’Connell R. A.; Loughrey J.; Sadiq G.; Davey R. J.; Roberts K. J. Influence of Solvent Composition on the Crystal Morphology and Structure of p-Aminobenzoic Acid Crystallized from Mixed Ethanol and Nitromethane Solutions. Cryst. Growth Des. 2017, 17, 4151–4161. 10.1021/acs.cgd.7b00425.

[ref29] Turner A.; Howell G.; Gall M.; Mulholland K.; Adlington N.; Tian Z.; Liu B.; Gong Q.; Yu W. Improved method for the manufacture of 3-[(1s)-1-imidazo[1,2-a]pyridin-6-ylethyl]-5-(1-methylpyrazol-4-yl)triazolo[4,5-b]pyrazine and polymorphic forms thereof. WO 2020053198 A1, 2020.

[ref30] Hammond R. B.; Pencheva K.; Roberts K. J. A Structural-Kinetic Approach to Model Face-Specific Solution/Crystal Surface Energy Associated with the Crystallization of Acetyl Salicylic Acid from Supersaturated Aqueous/Ethanol Solution. Cryst. Growth Des. 2006, 6, 1324–1334. 10.1021/cg0505618.

[ref31] Alharbi E. A.; Alyamani A. Y.; Kubicki D. J.; Uhl A. R.; Walder B. J.; Alanazi A. Q.; Luo J.; Burgos-Caminal A.; Albadri A.; Albrithen H.; Alotaibi M. H.; Moser J.-E.; Zakeeruddin S. M.; Giordano F.; Emsley L.; Grätzel M. Atomic-level passivation mechanism of ammonium salts enabling highly efficient perovskite solar cells. Nat. Commun. 2019, 10, 3008 10.1038/s41467-019-10985-5. 31285432 PMC6614363

[ref32] Zhou Y.; Wang J.; Xiao Y.; Wang T.; Huang X. The Effects of Polymorphism on Physicochemical Properties and Pharmacodynamics of Solid Drugs. Curr. Pharm. Des. 2018, 24, 2375–2382. 10.2174/1381612824666180515155425. 29766778

[ref33] Schick C. Differential scanning calorimetry (DSC) of semicrystalline polymers. Anal. Bioanal. Chem. 2009, 395, 1589–1611. 10.1007/s00216-009-3169-y. 19834693

